# Modified Dresden Technique With a Distal Locking Suture for Achilles Tendon Repair: Technique Tip

**DOI:** 10.1177/10711007241230987

**Published:** 2024-02-29

**Authors:** Kevan Kostynski, Amanda Vandewint, Amir Reza Vosoughi, Eva Gusnowski, Jacob Matz

**Affiliations:** 1 Faculty of Medicine, Dalhousie Medicine New Brunswick, Saint John, NB, Canada; 2 Canada East Foot & Ankle, Saint John, NB, Canada; 3 Horizon Health Network, Saint John, NB, Canada; 4 Bone and Joint Diseases Research Center, Department of Orthopedic Surgery, School of Medicine, Shiraz University of Medical Sciences, Shiraz, Iran; 5 Division of Orthopaedic Surgery, Department of Surgery, Dalhousie University/DMNB, Saint John, NB, Canada

**Keywords:** Achilles tendon rupture, Dresden technique, locking suture

## Abstract

**Graphical Abstract:**
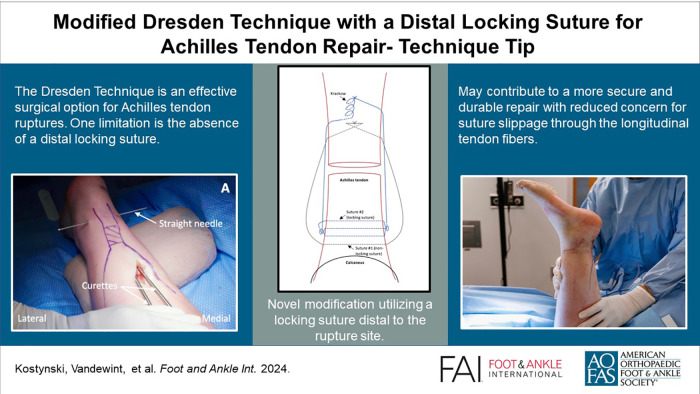
This is a visual representation of the abstract.

This is a visual representation of the abstract.

## Introduction

The Achilles tendon, the strongest and largest tendon in the human body, is one of the most frequently ruptured, and the optimal treatment approach for acute ruptures remains controversial.^8^ With operative management, minimally invasive surgery (MIS) is advantageous, decreasing infection, wound healing complications, ankle stiffness, and surgical time versus open repairs.^2^

**Figure 1.**
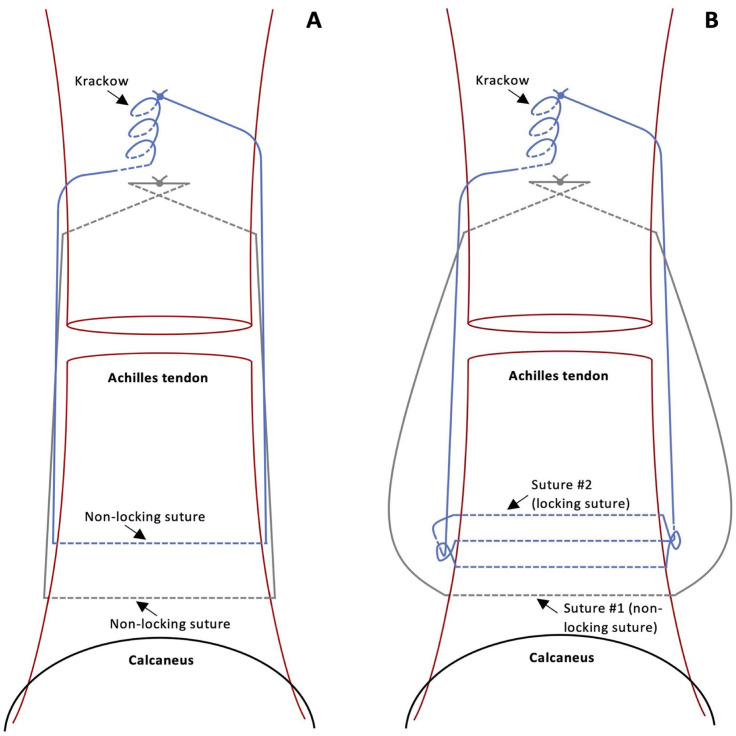
(A) Illustration demonstrating the position of the nonlocking sutures in the Achilles tendon according to the original Dresden technique. (B) Illustration demonstrating the position of suture 1 (nonlocking) and suture 2 (locking) in the Achilles tendon according to our modified Dresden technique. Solid lines denote suture thread superficial to the tendon whereas the interrupted lines denote suture thread passing through the tendon.

The Dresden technique (Figure 1A) has emerged as an effective MIS option.^1,5^ It involves passing sutures percutaneously through the distal Achilles tendon stump, retrieving them proximally, and tying them to the proximal tendon stump to complete the repair. This technique provides distinct advantages compared with other MIS options.^5^ First, an incision is made in a posteromedial location, minimizing the risk of sural nerve injury.^1,5^ Second, unlike other MIS techniques,^5^ surgical instruments are inserted in the plane between the crural fascia and paratenon, avoiding elevation of the paratenon from the Achilles tendon, thereby preserving maximal biology for healing.^1^ Finally, knots are tied proximal to the rupture site, reducing their prominence.^1^

One limitation of the Dresden technique is the absence of a distal locking suture, leaving potential for suture displacement through the distal tendon stump fibers and eventual rerupture.^3,4^ To optimize this, we present a novel modification using a locking suture distal to the Achilles tendon rupture site, delivering a more structurally secure and reliable repair (Figure 1B).

## Technique

**Figure 2.**
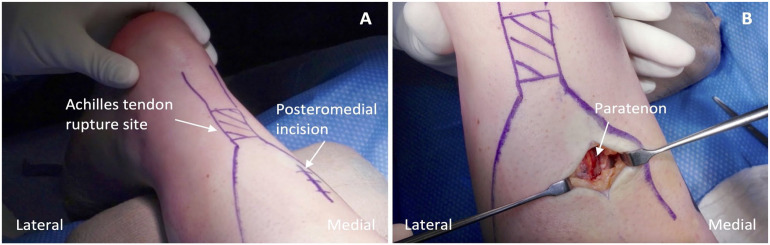
(A) Outline of the palpable gap marking the Achilles tendon rupture site and of the planned location for the posteromedial incision. (B) Appearance of the paratenon identified deep to the crural fascia.

For this procedure, general anesthesia is administered and the patient is secured in the semi-prone position.^7^ The Thompson test yields a positive result, representing loss of plantarflexion on the affected side (Supplemental Video 1). The patient’s leg is prepped and draped in a sterile fashion and a thigh tourniquet is inflated to 300 mm Hg. A 3-cm posteromedial incision is made 5 cm proximal to the rupture (Figure 2A). Dissection reveals the crural fascia, which is opened, exposing the Achilles tendon paratenon (Figure 2B). The paratenon is not opened, preserving tendon biology and the rupture hematoma.

**Figure 3.**
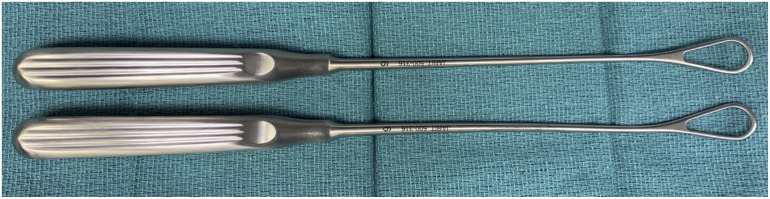
Two size 6 Integra Thomas Blunt Uterine Curettes (11").

**Figure 4.**
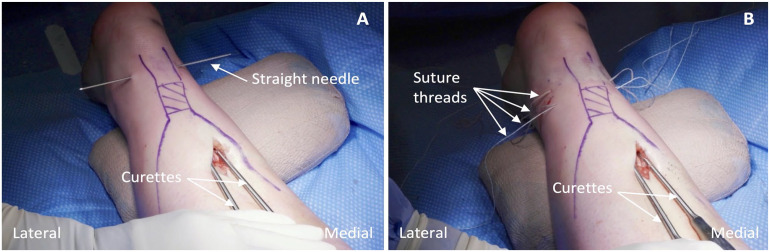
(A) Percutaneous passage of the first straight needle through the curette openings with the thickness of the distal Achilles tendon grasped in the process. (B) Placement of the four suture threads once inserted through the distal tendon stump.

We modified the Dresden technique by passing two size 6 Integra Thomas Blunt Uterine Curettes (11″; Central Infusion Alliance, Inc, Skokie, IL) from proximal to distal in the plane between the crural fascia and paratenon on both sides of the Achilles tendon (Figures 3 and 4A). The first curette is positioned laterally, approximately 1 cm proximal to the tendon’s insertion on the calcaneus. A second medial curette is positioned identically. Two strands of suture tape and two sutures with a looped end and a nonlooped end are required (PARS SutureTape Implant System; Arthrex, Naples, FL). Alternatively, this can be completed using four No. 1 Ethibond sutures (Ethicon, Sommerville, NJ), two of which require a slip knot tied at one end (Supplemental Video 2). A straight needle with a loop is used to pass the sutures. The first straight needle is passed medial to lateral through both curette openings, capturing the Achilles tendon. A second straight needle is inserted 5 mm proximal to the first in the same fashion. Suture 1 is threaded into the first needle and pulled through, spanning the tendon cross section. Suture 2 is then pulled similarly across the tendon. Sutures 3 and 4 are inserted in two positions proximal to suture 2, following the steps previously outlined, ensuring the looped ends are opposing each other (Figure 4B).

**Figure 5.**
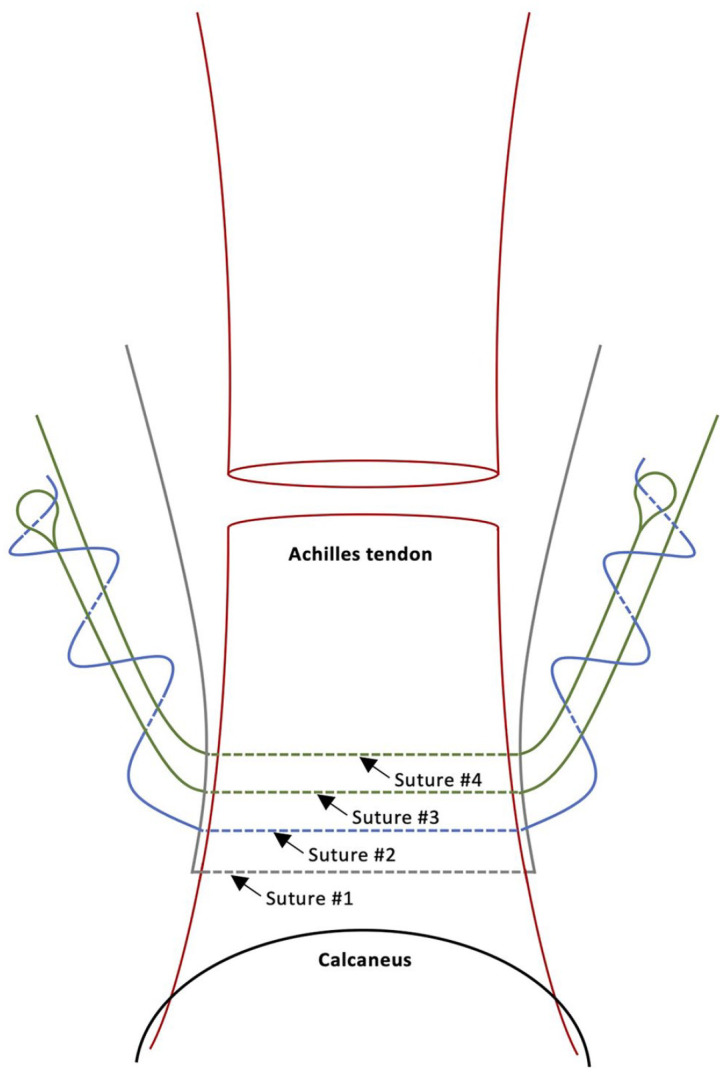
Illustration demonstrating the modified inclusion of the distal locking suture 2 going under and around the bundle of sutures 3 and 4 twice and through the loop on each side, locking suture 2 in the Achilles tendon. Solid lines denote suture thread superficial to the tendon whereas interrupted lines denote suture thread passing through the tendon. Figure based on: Arthrex. Achilles PARS SutureTape with Achilles Midsubstance SpeedBridge™️ Implant System – Surgical Technique. 2019. https://www.arthrex.com/resources/LT1-00100-EN/achilles-pars-suturetape-with-achilles-midsubstance-speedbridge-implant-system?referringteam=foot_and_ankle.

**Figure 6.**
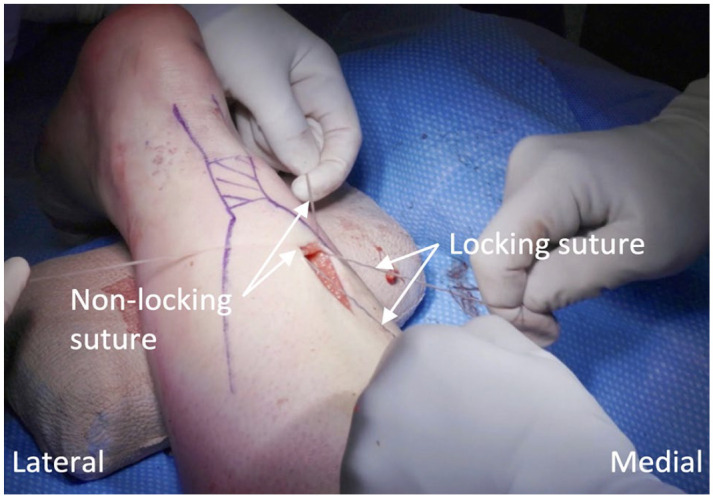
View of the two retained suture threads, suture 1 (nonlocking) and suture 2 (locking), anchored in the distal Achilles tendon stump.

The four suture threads are pulled through the incision by removing the curettes, then organized according to their original placement. The suture purchase within the tendon is tested by pulling the ends of each suture separately, ensuring a satisfactory plantarflexion response. The modified inclusion of a distal locking suture is performed by passing suture 2 under and around the bundle of sutures 3 and 4 twice, then through the suture loop on each side (Figure 5). Pulling on the nonlooped suture end on each side brings the suture through the tendon and out the opposing side, locking suture 2. The more distal suture 1 remains as a nonlocking suture. At this time, only suture 1 (nonlocking) and suture 2 (locking) remain anchored in the distal stump (Figure 6).

**Figure 7.**
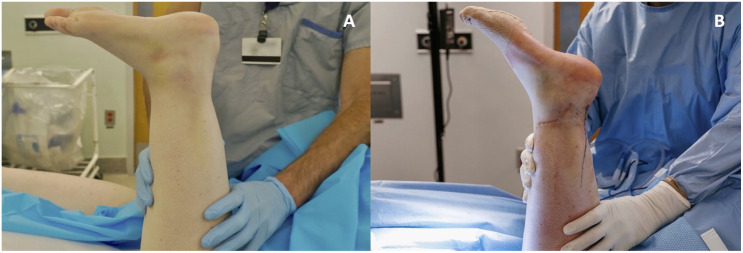
(A) Resting plantarflexion angle of the injured leg preoperatively. (B) Resting plantarflexion angle of the injured leg postoperatively.

Proximal fixation is achieved with the foot maintained in maximal plantarflexion, using a free curved needle to pass the medial and lateral ends of suture 1 across the tendon aponeurosis obliquely, in opposite directions creating a sling configuration. This sliding knot is tied, securing tension of the repair. Suture 2 is passed with a free curved needle through the aponeurosis using the Krackow technique^6^ and this knot is then tied, concluding reapproximation of the tendon ends. These knots are buried, the resting plantarflexion angle is checked, and the Thompson test is repeated to ensure appropriate plantarflexion (Figure 7A and B).

The procedure is completed by closing the crural fascia with 2-0 Vicryl and the subcutaneous layer with 3-0 Monocryl. The postoperative protocol from Patel and Kadakia^8^ is followed with no weightbearing in a splint for two weeks, then transfer into a walking boot as range of motion and weightbearing status progressively advance.

## Discussion

Repairing acute Achilles tendon ruptures with the Dresden technique preserves the paratenon, avoids prominent knots at the rupture site, and minimizes the risk of wound complications and sural nerve injuries.^1,5^ The weakness of this technique is the absence of a distal locking suture, leaving the potential for suture displacement through the distal tendon stump and rerupture.

The value of locking sutures in limited incision Achilles repairs was previously demonstrated by Demetracopoulos et al,^4^ whereby repairs with locking sutures showed greater construct strength. Recently, Chuckpaiwong et al^3^ found a nonlocking percutaneous repair to be structurally inferior to an open repair containing locking sutures. As such, Achilles tendon repair techniques that include locking sutures are advantageous, potentially resulting in increased resistance to gapping and improved repair durability.^3,4^

This modification of the Dresden technique uses generic curettes for suture passage, and two looped sutures to introduce a distal locking suture into the Achilles tendon. This addition enables surgeons to provide a secure Achilles tendon repair, reducing concern for suture slippage through the longitudinal tendon fibers.

## Supplemental Material

sj-pdf-1-fai-10.1177_10711007241230987 – Supplemental material for Modified Dresden Technique With a Distal Locking Suture for Achilles Tendon Repair: Technique Tip Supplemental material, sj-pdf-1-fai-10.1177_10711007241230987 for Modified Dresden Technique With a Distal Locking Suture for Achilles Tendon Repair: Technique Tip by Kevan Kostynski, Amanda Vandewint, Amir Reza Vosoughi, Eva Gusnowski and Jacob Matz in Foot & Ankle International
